# Violence Against Doctors by Patients and Their Relatives in Hospital Settings: A Narrative Review of Risk Factors, Communication Failures, and Prevention Strategies

**DOI:** 10.7759/cureus.112468

**Published:** 2026-07-11

**Authors:** Mohammed K Elbahi, Abdulgadir Elsunny Hamadelnil, Bahaeldein Mohammed, Awab Mohammed, Tibyan Abdalla Elniwayri Mohamed, Athambawa Abdul Azeez Fathima Zamharah, Sedigha Abdulla Mohamed Rooshenas, Alia Fathi Ibrahim Saleh

**Affiliations:** 1 Surgery, Faculty of Medicine, Al Neelain University, Khartoum, SDN; 2 General Surgery, Redsea University, Port Sudan, SDN; 3 Surgery, Prince Digna Referral Hospital, Port Sudan, SDN; 4 General Surgery, Prince Osman Digna Referral Hospital, Port Sudan, SDN; 5 Medicine and Surgery, University of El Imam El Mahdi, Kosti, SDN; 6 Medicine and Surgery, General Sir John Kotelawala Defence University Sri Lanka, Doha, QAT; 7 Medicine and Surgery, I.M. Sechenov First Moscow State Medical University, Doha, QAT; 8 Medicine and Surgery, Bahçeşehir University, Doha, QAT

**Keywords:** communication failure, patient aggression, patients’ relatives, prevention strategies, violence against doctors, workplace violence

## Abstract

Workplace violence against doctors has become an increasingly important concern in hospital settings, particularly in emergency departments and high-volume tertiary hospitals where clinical urgency, emotional distress, overcrowding, and resource limitations commonly intersect. Patients’ relatives and companions represent a particularly important group in this context because they often function as caregivers, advocates, financial decision-makers, and intermediaries between the patient and the healthcare system. Their aggressive reactions may be triggered by fear, uncertainty, poor prognosis, perceived delays, unmet expectations, or inadequate communication.

This narrative review synthesizes current evidence on violence against doctors by patients and their relatives in hospital settings, with emphasis on risk factors, communication failures, psychological and system-level drivers, consequences, and prevention strategies. A structured Scale for the Assessment of Narrative Review Articles (SANRA)-guided narrative methodology was used to review relevant literature from PubMed/MEDLINE, ScienceDirect, SpringerLink, BMJ Open, PubMed Central, Google Scholar, and institutional sources such as the World Health Organization. Included sources addressed workplace violence, patient or relative aggression, emergency department violence, doctor-patient communication, burnout, and mitigation strategies.

The evidence indicates that aggression toward doctors is usually multifactorial and can be understood through both organizational behavior and patient-provider conflict perspectives. From an organizational perspective, violence may arise when overcrowding, staff shortages, long waiting times, limited resources, weak security systems, and inadequate reporting mechanisms create a high-pressure clinical environment. From an interpersonal conflict perspective, aggression may develop when relatives’ expectations, emotional distress, and need for information are not aligned with clinical realities, triage priorities, or available hospital resources. Communication failure appears to be one of the most modifiable drivers of aggression, particularly when relatives receive inconsistent or insufficient information about diagnosis, prognosis, delays, or clinical priorities. The consequences of violence extend beyond immediate harm and include anxiety, burnout, reduced morale, defensive medical practice, disruption of team functioning, underreporting, and compromised patient safety.

Overall, violence against doctors by patients and their relatives should be approached as both an occupational safety issue and a healthcare quality problem. Effective prevention requires integrated hospital-level strategies, including communication training, structured family updates, visible triage explanations, waiting-time communication, environmental safety measures, incident reporting systems, leadership accountability, and post-incident support. Future research should examine patients’ relatives as a distinct group, particularly in emergency departments, tertiary hospitals, and resource-limited healthcare settings.

## Introduction and background

Workplace violence against healthcare workers has become a major concern within modern health systems, as it threatens staff safety, disrupts clinical care, and undermines the quality and continuity of healthcare delivery. Although violence may occur in different occupational settings, hospitals represent a particularly vulnerable environment because clinical encounters often take place under emotional distress, uncertainty, pain, fear, and high expectations from patients and their families. Healthcare professionals, especially doctors working in emergency departments, inpatient wards, and high-pressure hospital units, are frequently exposed to verbal abuse, threats, intimidation, and physical assault during routine clinical practice [1,2].

Current evidence indicates that violence against doctors is not an isolated interpersonal problem but a complex interaction between patient-related, physician-related, and system-level factors. Systematic reviews have shown that aggression toward physicians is associated with multiple determinants, including dissatisfaction with care, long waiting times, perceived delays in treatment, poor communication, unmet expectations, overcrowding, and limited institutional mechanisms for prevention and response [1,2]. In African healthcare settings, these challenges may be intensified by resource constraints, staff shortages, overcrowded facilities, and weak reporting systems, which can make both prevention and documentation of workplace violence more difficult [3].

For the purpose of this review, workplace violence was operationally defined as any incident in which doctors or healthcare workers are verbally abused, threatened, intimidated, psychologically harassed, or physically assaulted in circumstances related to their clinical work. This definition is consistent with international workplace violence frameworks that classify violence in healthcare as a spectrum ranging from verbal aggression and threats to physical assault [4]. The present review focuses specifically on Type II workplace violence, also described as client, patient, relative, companion, or visitor-on-worker violence, where the perpetrator is a recipient of care or someone emotionally or socially connected to the patient. This distinction is important because aggression from patients’ relatives differs from general workplace conflict; it often occurs during emotionally charged clinical encounters and is shaped by fear, uncertainty, perceived delay, unmet expectations, communication gaps, and dissatisfaction with hospital processes.

The scope of this review is intentionally focused on aggression involving patients’ relatives, companions, and visitors, and on communication and system-level contributors to such aggression. Behavioral-emergency presentations in which violence is primarily driven by acute intoxication, delirium, dementia, psychosis, severe agitation, or other patient-related clinical states are outside the main scope of this review. Similarly, the clinical management of violent behavioral emergencies, including verbal de-escalation for acutely agitated patients, chemical restraint, and physical restraint protocols, is not addressed in detail. This scoping distinction is important because patient-driven emergency violence and relative-driven aggression may share the same clinical environment but often differ in mechanisms, prevention priorities, and management strategies.

Patients’ relatives and companions represent an important but sometimes underemphasized source of aggression in hospital settings. Unlike patients themselves, relatives often experience the clinical encounter indirectly through anxiety, uncertainty, fear of deterioration, financial pressure, and limited understanding of disease severity or hospital processes. These emotional and informational gaps may contribute to hostile reactions, particularly when families perceive that the patient is not receiving timely attention or that doctors are not communicating clearly. Emergency departments are especially vulnerable because relatives frequently encounter long waiting times, unpredictable triage decisions, restricted access to clinical areas, and sudden changes in prognosis [2].

Communication failure is increasingly recognized as a central driver of conflict between doctors, patients, and relatives. Poor explanation of diagnosis, management plans, prognosis, waiting time, or reasons for clinical delay may create mistrust and intensify frustration. Conversely, clear, respectful, and empathic communication may reduce misunderstanding and improve acceptance of difficult clinical decisions. However, communication does not occur in isolation; doctors may be required to communicate under severe workload, limited resources, overcrowding, and emotional pressure, all of which can impair the quality of interaction and increase the risk of confrontation [2,4].

To move beyond descriptive synthesis, this review applies an integrated conceptual framework that combines organizational behavior theory with patient-provider conflict perspectives. Within this framework, aggression from patients’ relatives is understood as the product of four interacting domains: hospital system pressure, relatives’ emotional and cognitive response, communication quality, and institutional prevention capacity. Hospital system pressure includes overcrowding, long waiting times, staff shortages, resource limitations, unclear triage processes, and weak security systems. Relatives’ emotional and cognitive response includes fear, grief, uncertainty, mistrust, perceived injustice, and unrealistic expectations. Communication quality includes the clarity, consistency, empathy, and timing of information provided by doctors and healthcare teams. Institutional prevention capacity includes reporting systems, de-escalation protocols, staff support, environmental safety, and leadership accountability. This framework highlights that violence is not simply the result of poor communication or individual behavior; rather, communication failure acts as a modifiable pathway through which system pressures and relatives’ distress may escalate into aggression.

The consequences of violence against doctors extend beyond the immediate incident. Exposure to aggression can contribute to anxiety, burnout, reduced morale, defensive medical practice, absenteeism, and impaired team performance. At the organizational level, repeated violence may weaken trust between healthcare workers and the community, reduce staff retention, and compromise patient safety by disrupting clinical workflow and decision-making. These consequences highlight the need to view workplace violence not only as a staff welfare issue but also as a patient safety and health system quality problem [1,4].

Despite growing international attention, much of the literature on workplace violence in healthcare discusses patients and relatives together, with less focused synthesis on the specific role of patients’ relatives as perpetrators or contributors to aggressive behavior. In addition, many studies describe prevalence and risk factors without sufficiently integrating communication failures, healthcare system challenges, and prevention strategies into a single practical framework. This gap is particularly relevant to resource-limited hospital settings, where overcrowding, delays, and limited communication infrastructure may increase the likelihood of conflict [2,3].

A key knowledge gap is that many existing studies measure the prevalence of workplace violence or list risk factors without clearly separating aggression from patients from aggression from relatives, companions, or visitors. In addition, limited attention has been given to how communication failure functions as a pathway between system-level strain and relatives’ aggressive reactions. This gap is particularly relevant in resource-limited hospitals, where overcrowding, delays, and limited staffing may intensify relatives’ frustration while reducing doctors’ ability to provide repeated, structured communication. Therefore, the added value of this review lies not in re-establishing that workplace violence exists, but in integrating evidence on relatives’ aggression, communication failure, organizational behavior, patient-provider conflict, and hospital-level prevention into a single clinically applicable framework.

This narrative review aims to synthesize current evidence on violence against doctors by patients and their relatives in hospital settings, with particular focus on risk factors, communication failures, psychological and system-level drivers, consequences for healthcare workers and organizations, and practical prevention strategies. By integrating evidence on patients’ relatives, doctor-patient communication, organizational behavior, patient-provider conflict, and hospital-level interventions, this review seeks to provide a clinically relevant conceptual framework for understanding and reducing aggression toward doctors across diverse healthcare settings.

## Review

Methodology

This study was conducted as a Scale for the Assessment of Narrative Review Articles (SANRA)-guided narrative review. The SANRA was used to support transparent reporting, clarity of aims, appropriate referencing, scientific reasoning, and balanced presentation of the literature. SANRA was not used as a systematic review methodology, database screening method, or formal quality assessment tool. The review was designed to provide a critical narrative synthesis of current evidence on violence against doctors by patients and their relatives in hospital settings, with emphasis on risk factors, communication failures, psychological and system-level drivers, consequences, and prevention strategies.

Because the objective was to develop a conceptual and clinically relevant synthesis rather than to estimate pooled prevalence or effect sizes, a meta-analysis was not performed. The review process included a structured literature search, transparent selection considerations, narrative information extraction, relevance appraisal, and thematic synthesis. This approach was selected to maintain the flexibility of a narrative review while improving transparency and reproducibility of interpretation.

Search Strategy

A structured and comprehensive search was conducted to identify relevant literature addressing workplace violence, patient aggression, aggression from patients’ relatives, and violence against doctors and healthcare workers in hospital settings. Major academic databases and scholarly platforms, including PubMed/MEDLINE, ScienceDirect, SpringerLink, BMJ Open, PubMed Central, Google Scholar, and relevant institutional sources such as the World Health Organization, were searched. The search strategy is summarized in Table 1.

**Table 1:** Summary of the literature search strategy Search strategy used to identify relevant literature on workplace violence, patient aggression, relatives’ aggression, communication failures, and prevention strategies.

Database or Source	Search Terms Used	Limits or Filters	Purpose of Search
PubMed/MEDLINE	“workplace violence” AND “doctors”	English-language articles; hospital or healthcare settings	To identify peer-reviewed studies on workplace violence against doctors and healthcare workers
PubMed/MEDLINE	“patient aggression” AND “physicians”	English-language articles; hospital-based studies	To identify studies focusing on aggression and violence against physicians
PubMed/MEDLINE	“emergency department violence” AND “healthcare workers”	English-language articles; emergency care settings	To identify studies on violence in emergency departments
ScienceDirect	“workplace violence” AND “healthcare professionals”	Review articles and original studies	To identify systematic reviews and narrative evidence on workplace violence in healthcare
ScienceDirect	“patient aggression” AND “physicians” AND “risk factors”	Review articles and hospital-based studies	To identify evidence on risk factors, consequences, and prevention of patient aggression
SpringerLink	“patient aggression” AND “physicians” AND “hospital interventions”	English-language articles	To identify studies on hospital-level prevention and management strategies
BMJ Open	“violence against physicians” AND “tertiary hospital”	Observational and cross-sectional studies	To identify hospital-based prevalence and risk factor studies
PubMed Central	“patients’ relatives” AND “violence” AND “doctors”	Full-text articles	To identify evidence related to relatives, companions, or visitors as contributors to aggression
Google Scholar	“doctor-patient communication” AND “violent behavior” AND “healthcare settings”	Relevant scholarly articles	To identify studies linking communication failures with violent behavior
World Health Organization	“preventing violence against health workers”	Institutional guidance	To identify global guidance and prevention recommendations

Search terms included combinations of keywords and phrases related to workplace violence, violence against doctors, patient aggression, patients’ relatives, patient companions, visitors, emergency department violence, healthcare workers, communication failure, doctor-patient communication, waiting time, burnout, and prevention strategies. Boolean operators such as AND and OR were used to refine the search. In addition, reference lists of selected articles were manually screened to identify further relevant studies.

Selection Considerations

Selection considerations were used to identify literature relevant to the aims of this narrative review. The inclusion and exclusion criteria used to guide source selection are presented in Table 2.

**Table 2:** Eligibility criteria for study selection Criteria used to determine the inclusion and exclusion of studies in this narrative review.

Category	Criterion
Inclusion	Studies addressing verbal abuse, threats, intimidation, physical assault, or other forms of aggression against doctors or healthcare workers
Inclusion	Studies conducted in hospital, emergency department, tertiary care, or other healthcare settings
Inclusion	Articles examining patients, patients’ relatives, companions, or visitors as perpetrators or contributors to aggression
Inclusion	Studies exploring communication failures, dissatisfaction with care, waiting times, or unmet expectations as contributors to workplace violence
Inclusion	Studies identifying risk factors, prevalence, consequences, or prevention strategies related to violence against healthcare workers
Inclusion	Systematic reviews, narrative reviews, observational studies, cross-sectional studies, qualitative studies, mixed-methods studies, and evidence-based institutional reports
Inclusion	Studies from low- and middle-income countries, African healthcare settings, emergency departments, and resource-limited systems where relevant to the review objective
Exclusion	Articles focused on violence unrelated to healthcare settings
Exclusion	Studies addressing occupational violence not involving patients, relatives, companions, or visitors
Exclusion	Articles focused exclusively on non-hospital workplace aggression without relevance to healthcare practice
Exclusion	Highly specialized studies without broader relevance to workplace violence against doctors or healthcare workers
Exclusion	Non-English articles
Exclusion	Conference abstracts without accessible full text or sufficient methodological detail
Exclusion	Opinion pieces or commentaries without supporting evidence

Articles were considered for inclusion if they addressed verbal abuse, threats, intimidation, physical assault, or other forms of aggression against doctors or healthcare workers in hospital or emergency care settings. Studies were also considered if they examined patients, patients’ relatives, companions, or visitors as perpetrators or contributors to aggression; explored communication failures or dissatisfaction with care; identified risk factors for workplace violence; assessed consequences for healthcare workers or organizations; or proposed prevention and mitigation strategies.

The review prioritized systematic reviews, narrative reviews, observational studies, qualitative studies, mixed-methods studies, and evidence-based institutional reports. Particular attention was given to studies from emergency departments, tertiary hospitals, low- and middle-income countries, African healthcare settings, and resource-limited systems because of their relevance to hospital-based practice.

Articles were excluded if they focused on violence unrelated to healthcare settings, occupational violence not involving patients or relatives, non-hospital workplace aggression, or highly specialized contexts without broader clinical relevance. Non-English articles, conference abstracts without accessible full text, and opinion pieces without supporting evidence were also excluded.

Information Extraction

A structured narrative information extraction process was used to collect key concepts from selected sources. Extracted information included study objectives, design, country or setting, study population, type of violence reported, perpetrator category, risk factors, communication-related contributors, consequences for doctors and healthcare teams, and proposed prevention strategies. This process was used to support thematic organization rather than quantitative pooling.

Specific attention was given to evidence describing aggression from patients’ relatives, companions, or visitors, as this represents a central focus of the review. Information related to system-level contributors, including overcrowding, long waiting times, staffing shortages, lack of resources, inadequate security, and weak reporting systems, was also extracted. Communication-related factors such as poor explanation, limited empathy, insufficient information sharing, perceived disrespect, and dissatisfaction with care were recorded because of their relevance to the conceptual framework of the review.

Relevance Appraisal

Because this was a narrative review rather than a systematic review, formal risk-of-bias assessment, quality scoring, and meta-analysis were not performed. Instead, included sources were appraised narratively for relevance to the review question, methodological clarity, study setting, population, and contribution to the conceptual synthesis. Greater emphasis was placed on peer-reviewed systematic reviews, hospital-based observational studies, qualitative or mixed-methods studies, and sources that directly addressed patients’ relatives, communication failures, workplace violence mechanisms, or prevention strategies.

Evidence from systematic reviews and hospital-based studies was used to support general patterns regarding prevalence, risk factors, and consequences, while qualitative and mixed-methods studies were used to explain mechanisms, context, and interpersonal dynamics. Non-peer-reviewed sources were used only when they provided relevant contextual or institutional guidance and were not treated as primary evidence. This approach was used to maintain consistency with the narrative review design while avoiding overstatement of methodological rigor.

Synthesis of Findings

A narrative thematic synthesis was used to integrate findings from the selected literature. Evidence was organized into major domains, including the epidemiology of workplace violence, high-risk hospital settings, aggression from patients’ relatives and companions, communication failures, psychological and emotional drivers, system-level contributors, consequences for doctors and healthcare organizations, and prevention strategies.

The synthesis aimed to identify recurring patterns across different healthcare systems while highlighting factors relevant to resource-limited hospital environments. Rather than presenting workplace violence as a single-cause phenomenon, the review examined how relatives’ emotional distress, unmet expectations, communication gaps, overcrowding, delays in care, and weak institutional prevention systems interact to increase the risk of aggression toward doctors.

By applying a SANRA-guided narrative approach, this review provides a transparent and clinically relevant synthesis of violence against doctors by patients and their relatives in hospital settings while avoiding claims of systematic review methodology.

To reduce duplication of existing reviews, primary hospital-based studies were used to complement broader review evidence wherever available. Systematic and narrative reviews were used to establish general patterns, while primary studies from emergency departments and tertiary hospitals were used to provide setting-specific data, perpetrator patterns, and context-specific mechanisms. The added conceptual framework was used to integrate these findings rather than reproduce prior syntheses.

Epidemiology of workplace violence against doctors

Workplace violence in healthcare is widely recognized as a global occupational and public health concern, affecting healthcare workers across different systems, specialties, and levels of care. Reported prevalence varies substantially because studies differ in definitions of violence, reporting systems, study populations, clinical settings, and recall periods. A systematic review focused on patient aggression and violence against physicians included 104 studies and found that prevalence was generally higher in developing countries and among younger physicians, with risk factors operating at patient, patient-physician interaction, hospital, and societal levels [2]. A broader systematic review of workplace violence against healthcare professionals also found that violence most commonly occurred in psychiatric departments, emergency services, outpatient or waiting areas, and geriatric units, with verbal abuse, psychological violence, physical assault, and sexual abuse being the main forms reported [1].

Emergency departments are particularly high-risk areas because they combine clinical urgency, overcrowding, long waiting times, unpredictable patient behavior, and frequent interaction with distressed relatives. In the United States, a nationally representative analysis of emergency department-treated occupational injuries from 2015 to 2017 estimated approximately 1.14 million injuries among healthcare workers, with intentional injuries by another person accounting for 15% of these healthcare-related injuries [5]. In Egypt, a cross-sectional study from the emergency departments of Ain Shams University Hospitals reported that verbal violence affected 86.1% of participating healthcare workers, followed by sexual violence in 48.1% and physical violence in 34.3% [6]. Patient companions were the most frequent perpetrators across all forms of violence, accounting for 80% of verbal violence, 86.5% of physical violence, and 73.1% of sexual violence incidents [6].

Evidence from African healthcare settings also demonstrates wide variation in reported prevalence. A systematic review of workplace violence against healthcare workers in Africa included 22 peer-reviewed studies with 8,065 respondents and reported overall workplace violence prevalence ranging from 9% to 100% across included studies. The highest reported ranges were from South Africa, 54% to 100%, and Egypt, 59.7% to 86.1%. By type of violence, verbal abuse was reported in 73%, emotional violence in 46%, and physical violence in 27% of included evidence [3]. These estimates show that workplace violence is not only present across African healthcare settings but also varies markedly according to country, setting, professional group, and violence definition.

Hospital-based studies from South Asia further illustrate the burden of violence against doctors in high-volume hospital settings. A facility-based cross-sectional study from Bangladesh reported workplace violence against physicians in a public tertiary care hospital [7]. In Bangladeshi tertiary care hospitals, 12.3% of doctors reported exposure to physical violence during the previous 12 months [8]. Among victims of physical violence, patients’ relatives were the main perpetrators in 72% of cases, followed by visitors in 24% and patients in 4% [8]. The same study reported that 26% of physically assaulted doctors were injured, 72% missed workdays or took sick leave, and 72% did not receive support from hospital officials after the incident [8]. These figures support the importance of examining relatives separately from patients when studying violence against doctors.

Underreporting is a major limitation in interpreting all prevalence estimates. Some healthcare workers do not formally report incidents because they view violence as part of routine clinical work, believe that reporting will not lead to meaningful action, fear negative consequences, or lack access to simple reporting systems. This reporting gap can substantially underestimate the true burden of workplace violence. For example, one systematic review noted that only around 15% of workplace violence cases were reported to police or public security authorities [1]. In the Bangladeshi physical-violence study, only 6% of incidents were reported to police, while 35% of victims reported that no action was taken after the incident [8]. Therefore, every prevalence figure should be interpreted in light of definitional differences, recall periods, reporting culture, and the availability of institutional reporting mechanisms.

Overall, the epidemiology of workplace violence against doctors demonstrates a consistent pattern: violence is common, frequently underreported, and concentrated in settings where clinical urgency, emotional stress, overcrowding, limited resources, and distressed relatives intersect. However, because available studies vary in design, definitions, populations, and reporting periods, the estimates should be interpreted descriptively rather than pooled. Selected quantitative findings from key included studies are summarized in Table 3.

**Table 3:** Quantitative summary of workplace violence findings from included studies Selected quantitative findings are summarized descriptively because definitions, populations, settings, and reporting periods varied across studies.

Study	Country/Setting	Study Design	Population	Reporting Period	Key Quantitative Findings
Carey and Hendricks [5]	United States; emergency department data	Nationally representative analysis	Healthcare workers in emergency department settings	2015-2017	Provided nationally representative estimates of workplace violence events in emergency department settings, highlighting emergency departments as a high-risk environment for violence against healthcare workers
Assil et al. [6]	Egypt; Ain Shams University Hospitals' emergency departments	Cross-sectional study	Emergency department healthcare workers	Study-defined reporting period	Reported workplace violence in emergency departments and identified verbal violence as a common form of aggression among exposed healthcare workers
Hasan et al. [7]	Bangladesh; public tertiary care hospital	Facility-based cross-sectional study	Physicians	12 months	Reported violence against physicians in a tertiary hospital setting, including verbal and physical forms of violence, and identified workplace and system-level factors associated with exposure
Shahjalal et al. [8]	Bangladesh; tertiary care hospitals	Cross-sectional study	Doctors	Study-defined reporting period	Focused specifically on physical violence against doctors and identified determinants associated with physical assault in tertiary care hospitals
Grover et al. [9]	India; tertiary care hospital	Cross-sectional study	Doctors	Study-defined reporting period	Reported workplace violence against doctors in a tertiary care setting, including verbal abuse, threats, and physical violence
Bingöl and İnce [10]	Emergency departments; relatives’ perspective	Cross-sectional/descriptive study	Patients’ relatives	Study-defined reporting period	Identified relatives’ perspectives on contributors to violence, including waiting time, overcrowding, poor communication, and dissatisfaction with care

Role of patients’ relatives and companions in aggression

Patients’ relatives and companions play a central role in the dynamics of hospital-based violence because they often experience the clinical encounter through emotional distress, uncertainty, and fear rather than direct clinical assessment. In many hospital settings, relatives are responsible for transporting patients, providing information, purchasing medications or investigations, making financial decisions, and communicating with doctors on behalf of the patient. This close involvement can make relatives highly invested in the outcome of care, but it can also increase the risk of frustration when communication is limited, waiting times are prolonged, or the patient’s condition deteriorates unexpectedly [10].

Unlike aggression from patients, which may sometimes be influenced by pain, intoxication, confusion, psychiatric illness, or acute physiological distress, aggression from relatives is often driven by emotional and situational factors. These include anxiety about prognosis, fear of death or disability, anger about perceived delay, dissatisfaction with explanations, financial pressure, and mistrust of the healthcare system. Relatives may interpret waiting, triage decisions, or limited bedside access as neglect, even when these actions are clinically justified. This mismatch between clinical reasoning and family perception can create conflict, especially in emergency and high-acuity settings [10].

Emergency departments are particularly vulnerable to aggression from relatives because they combine uncertainty, overcrowding, time pressure, and emotionally charged clinical presentations. Relatives may arrive expecting immediate treatment, rapid diagnosis, or constant updates, while doctors are required to prioritize patients according to clinical urgency. When this process is not clearly explained, relatives may perceive triage as unfair or delayed care as negligence. Evidence from studies examining relatives’ perspectives suggests that poor communication, insufficient information, overcrowding, and prolonged waiting times are among the most important perceived drivers of violent behavior in emergency departments [10].

Psychological responses among patients and relatives may also contribute to hostility toward doctors. Feelings of helplessness, anger, disappointment, or perceived injustice can intensify when clinical outcomes are poor or when expectations are not met. Some studies have explored negative emotional perceptions toward doctors, including resentment and satisfaction at doctors’ misfortune, reflecting deeper problems of mistrust and damaged doctor-patient relationships in certain healthcare environments [11]. Although such findings should not be generalized to all relatives, they highlight the importance of rebuilding trust, transparency, and respectful communication between healthcare workers and the communities they serve.

Aggression from relatives is also shaped by the structure of the hospital environment. Crowded waiting areas, lack of privacy, poor signage, limited staff availability, and absence of clear information channels can increase tension before any direct doctor-relative interaction occurs. In many resource-limited hospitals, relatives may also be required to obtain medications, blood products, imaging, or supplies from outside the hospital, creating additional stress and delays. When these system failures are not explained, doctors may become the visible target of anger despite having limited control over the underlying resource constraints [3,10].

The role of relatives should therefore be understood within a broader social and institutional context. Relatives are not only visitors; in many healthcare systems, they function as caregivers, advocates, decision-makers, and financial supporters. Their aggression may arise when this caregiving role becomes overwhelmed by fear, poor communication, uncertainty, and perceived system failure. Recognizing this complexity is essential for designing prevention strategies that do not rely only on security responses, but also include structured communication, family information systems, waiting-time explanations, and early conflict de-escalation pathways [10,12].

Risk factors and communication failures

Violence against doctors by patients and their relatives is usually multifactorial, arising from the interaction between individual frustration, emotional distress, communication breakdown, and healthcare system limitations. Current evidence suggests that aggression is rarely caused by a single event; rather, it often develops when relatives experience uncertainty, prolonged waiting, poor explanations, perceived disrespect, or dissatisfaction with the care process [12,13]. In this context, doctors may become the immediate target of anger even when the underlying trigger is related to broader institutional constraints such as overcrowding, staff shortages, lack of beds, or limited access to investigations and medications.

Communication failure is one of the most consistently reported contributors to aggression in healthcare settings. When doctors do not clearly explain the diagnosis, treatment plan, prognosis, expected waiting time, or reasons for delay, patients and relatives may interpret the situation as neglect or lack of concern. This perception can be particularly strong in emergency departments, where relatives often expect immediate intervention and may not understand triage priorities. Even when clinical decisions are appropriate, inadequate communication can create mistrust and increase the likelihood of confrontation [10,13].

The quality of doctor-relative communication is influenced by both interpersonal and system-level factors. At the interpersonal level, relatives may respond negatively to communication that appears rushed, dismissive, emotionally detached, or overly technical. A lack of empathy, poor listening, and failure to acknowledge family concerns may intensify anger, especially when the patient’s condition is serious or deteriorating. At the system level, high patient load, fatigue, repeated interruptions, and limited consultation time can reduce doctors’ ability to communicate effectively, creating a cycle in which workload contributes to poor communication and poor communication increases the risk of aggression [13,14].

Long waiting times and perceived delays in care are also major drivers of violence. Relatives may not distinguish between unavoidable system delays and clinician-related delays, particularly when they receive little information about triage, bed availability, investigation results, or operative scheduling. Waiting becomes more difficult to tolerate when families are anxious, the patient is in pain, or the clinical condition appears urgent. Peer-reviewed evidence from emergency department and relatives’ perspective studies has linked waiting times, overcrowding, poor communication, and unmet expectations with increased aggression toward healthcare workers [10]. A recent media report similarly described waiting times and refusal of requests as contextual triggers for violence in primary care, but this was treated as illustrative background rather than primary evidence [15].

Unmet expectations are another important risk factor. Patients and relatives may arrive at hospital expecting rapid diagnosis, immediate treatment, guaranteed recovery, or direct access to senior doctors. When these expectations are not met, dissatisfaction may develop, especially if the limitations of the healthcare system are not explained. In resource-limited settings, the gap between what relatives expect and what hospitals can realistically provide may be wide. Shortages of medications, imaging delays, lack of available beds, and financial barriers can all contribute to frustration, yet doctors may be blamed because they are the most visible representatives of the system [3,13].

Perceived disrespect and loss of trust further increase the risk of aggression. Some relatives may interpret clinical prioritization, restricted access, or brief communication as intentional disregard. This is especially likely when there is no structured system for regular updates or when information is delivered inconsistently by different members of the healthcare team. Conflicting explanations from staff can create confusion and suspicion, while absence of clear documentation or communication pathways may weaken confidence in clinical decisions. Trust is therefore strongly linked not only to what doctors do clinically, but also to how decisions are explained and how reliably information is shared [12,13].

Healthcare system pressures can magnify all these risks. Overcrowding, understaffing, weak security presence, lack of violence reporting systems, and poor hospital infrastructure create an environment in which conflict is more likely to escalate. In such settings, even minor communication gaps may trigger aggressive reactions because relatives are already frustrated by long queues, unclear processes, and limited resources. These factors show that preventing violence requires more than asking individual doctors to communicate better; it also requires institutional systems that support communication, reduce avoidable delays, and provide clear mechanisms for conflict prevention and response [4,13].

Overall, the literature indicates that communication failures, waiting times, unmet expectations, and system limitations are closely interconnected risk factors for aggression against doctors. Effective prevention, therefore, requires a combined approach that strengthens doctors’ communication skills while also improving hospital-level processes such as triage explanation, family updates, complaint handling, security support, and reporting systems. This integrated approach is particularly important in emergency and resource-limited settings, where relatives’ distress and system pressures frequently overlap [4,10,13].

Consequences for doctors, healthcare teams, and organizations

The consequences of violence against doctors extend well beyond the immediate physical or verbal incident. Exposure to aggression can produce psychological distress, fear, anger, reduced confidence, and a persistent sense of vulnerability within the clinical environment. Doctors who repeatedly experience threats, insults, intimidation, or assault may begin to anticipate conflict during patient encounters, particularly in emergency departments and high-pressure hospital units. This can alter communication patterns, reduce job satisfaction, and weaken the therapeutic relationship between doctors, patients, and relatives [16,17].

At the individual level, workplace violence is strongly linked to emotional exhaustion and burnout. Doctors and emergency department staff exposed to aggression from patients and relatives may experience anxiety, sleep disturbance, reduced motivation, and symptoms of occupational stress. Over time, repeated exposure may contribute to depersonalization, reduced empathy, and withdrawal from direct communication with patients or relatives. This is particularly concerning because communication avoidance may itself worsen dissatisfaction and increase the likelihood of future conflict, creating a harmful cycle between violence, burnout, and poorer doctor-relative interaction [17].

Violence may also contribute to changes in clinical decision-making, although the available evidence is largely qualitative and should be interpreted cautiously. Doctors who feel unsafe or unsupported may become more defensive in practice, may avoid difficult conversations, or may request additional investigations partly to reduce confrontation rather than solely because of clinical need. In some cases, fear of relatives’ reactions may influence how prognosis, death, complications, or treatment limitations are communicated. These effects may increase workload, reduce confidence in clinical communication, and contribute to staff dissatisfaction, intent to leave, and workforce attrition, particularly when institutional support after violent incidents is perceived as inadequate [16].

At the team level, aggression disrupts workflow and weakens morale. Violent incidents can interrupt clinical tasks, delay treatment for other patients, and divert staff attention from patient care to conflict management. When one doctor or healthcare worker is attacked, the psychological effect often spreads to the wider team, producing fear, frustration, and reduced sense of workplace safety. Qualitative evidence indicates that patient aggression and violence against physicians can negatively affect team communication, organizational trust, and the overall working climate [16].

The organizational consequences are equally important. Hospitals with frequent violence may experience increased absenteeism, staff turnover, reduced productivity, and difficulty retaining experienced healthcare workers. Repeated incidents may also damage the hospital’s reputation and weaken community trust. If healthcare workers believe that violent incidents are ignored, normalized, or poorly managed, they may become less likely to report future events. This underreporting prevents accurate measurement of the problem and limits the ability of hospital leadership to design effective prevention strategies [16,17].

Violence against doctors should therefore be understood as both an occupational safety issue and a healthcare quality problem. It affects not only the well-being of doctors but also team performance, communication quality, patient flow, and patient safety. When doctors work in an environment where aggression is expected or tolerated, the ability to provide calm, compassionate, and timely care is compromised. Addressing workplace violence is therefore essential for protecting healthcare workers, maintaining functional clinical teams, and improving the reliability of hospital care [16,17].

Prevention and mitigation strategies

Preventing violence against doctors by patients and their relatives requires a coordinated approach that combines communication improvement, environmental safety, reporting systems, staff training, and institutional accountability. Evidence suggests that isolated interventions are unlikely to be effective when violence is driven by multiple interacting factors such as long waiting times, overcrowding, poor information flow, emotional distress, and weak security systems. Therefore, prevention should be framed as a hospital-level safety strategy rather than as the responsibility of individual doctors alone [18,19].

Communication-based interventions are frequently recommended as part of workplace violence prevention, although the strength of evidence varies across intervention types. Evidence from rapid reviews suggests that de-escalation training may improve staff knowledge, confidence, and preparedness, but high-quality evidence demonstrating a consistent reduction in actual assault rates remains limited. Expert-consensus work also supports risk communication, empathy, conflict recognition, and de-escalation as feasible hospital-level interventions, particularly in emergency departments where relatives often require repeated updates about triage decisions, expected delays, investigation results, and changes in prognosis. Therefore, communication training should be viewed as a practical and commonly recommended component of prevention, but not as a standalone intervention with definitive effectiveness [18,19].

The prevention evidence should therefore be interpreted according to its strength. Some interventions, such as de-escalation training, structured communication approaches, and environmental safety measures, are supported by rapid-review evidence suggesting improvements in staff preparedness, confidence, and perceived safety, but evidence for consistent reductions in actual assault rates remains limited. Other measures, including hospital-wide policy development, leadership accountability, visitor management, and structured reporting systems, are commonly supported by expert consensus and institutional guidance rather than definitive effectiveness trials. For this reason, prevention strategies should be presented as components of a layered risk-reduction framework rather than as individually proven interventions.

Hospitals should also develop clear systems for family information and waiting-time communication. Many aggressive incidents arise when relatives feel ignored or uninformed, even when clinical care is ongoing. Regular updates, visible triage explanations, designated communication points, and clear signage may reduce frustration before it escalates into verbal or physical aggression. In high-volume settings, assigning a trained staff member to provide non-confidential process updates may help reduce pressure on doctors while maintaining communication with families [18,19].

Environmental and security measures are also essential, particularly in emergency departments where clinical uncertainty, overcrowding, and high emotional tension increase the risk of escalation. Practical emergency department-applicable measures include the presence of trained security personnel, controlled access to clinical areas, visitor limitation during high-risk periods, panic alarms, clear lockdown procedures, and rapid response pathways for escalating violent behavior. Hospitals may also consider patient flagging or alert systems for individuals with a documented history of violence, provided that these systems are used ethically, reviewed regularly, and do not compromise necessary clinical care. Security protocols should clearly define when staff should call for assistance, how violent incidents are escalated, and how doctors and nurses are protected during active threats. However, security should not be used as the only solution. A purely reactive model may reduce immediate harm but does not address the underlying causes of aggression. The most effective approaches combine early identification of risk, staff training, structured communication, environmental design, and rapid response protocols [19].

Incident reporting systems are another key component of violence prevention. Underreporting remains a major barrier because many doctors consider verbal abuse and threats as part of routine work or believe that reporting will not lead to meaningful action. Hospitals should establish simple, confidential, and non-punitive reporting systems that capture verbal abuse, threats, physical assault, and near-miss events. Regular analysis of these reports can help identify high-risk times, locations, departments, and triggers, allowing targeted interventions instead of generalized policies [18,19].

Organizational leadership plays a central role in sustaining prevention efforts. Hospital administrations should communicate a clear zero-tolerance stance toward violence while also ensuring that staff receive practical support after incidents. Post-incident debriefing, psychological support, legal guidance, and administrative follow-up help reinforce trust between healthcare workers and the institution. Without visible organizational response, doctors may feel abandoned, reporting rates may decline, and violence may become normalized within the workplace [18].

Public and community-level interventions are also important, especially where mistrust between communities and healthcare systems contributes to aggression. Awareness campaigns can clarify the role of triage, explain why delays occur, and emphasize that violence disrupts care for all patients. Community education should also reinforce that doctors often operate within system limitations and should not be personally blamed for shortages of beds, medications, investigations, or staff. Such interventions may help reduce unrealistic expectations and improve the relationship between hospitals and the communities they serve [18,19].

Overall, prevention strategies should address both immediate safety and upstream drivers of aggression. A practical hospital framework should include communication training, structured family updates, waiting-time information, environmental safety measures, accessible reporting systems, rapid response protocols, and leadership accountability. In resource-limited hospitals, low-cost options such as triage explanation posters, designated family update times, staff de-escalation training, and simple incident reporting forms may be plausible and feasible starting points, but their effectiveness, cost, and implementation acceptability require local evaluation before they can be recommended as proven interventions. Reducing violence against doctors therefore requires an integrated system that protects healthcare workers while improving communication, trust, and patient-centered care [18,19].

Discussion

Violence against doctors by patients and their relatives represents a complex healthcare challenge shaped by emotional distress, communication gaps, and institutional limitations. The findings synthesized in this review suggest that aggression in hospital settings should not be understood only as an isolated behavioral problem, but as the visible outcome of multiple interacting pressures within the healthcare encounter. Patients and relatives often arrive at hospitals during moments of fear, pain, uncertainty, and high expectation, while doctors are required to make rapid clinical decisions under workload, resource constraints, and time pressure. When these realities collide without effective communication and institutional support, conflict becomes more likely [1,2].

A consistent theme across the literature is that emergency departments and high-volume tertiary hospitals are particularly vulnerable to violence. These settings combine overcrowding, long waiting times, unpredictable clinical severity, and emotionally charged interactions with relatives. In such environments, relatives may perceive triage delays, limited access, or lack of repeated updates as neglect, even when clinical prioritization is appropriate. This highlights the importance of explaining hospital processes clearly, especially triage, waiting time, investigation delays, bed availability, and reasons for clinical prioritization [5,6,10].

The role of patients’ relatives is especially important because relatives often act as caregivers, decision-makers, financial supporters, and advocates. Their aggression may arise from fear of losing the patient, poor understanding of disease severity, perceived lack of attention, or frustration with system delays. Unlike patient-related aggression, which may sometimes be influenced by pain, confusion, psychiatric illness, or intoxication, relatives’ aggression is more commonly linked to emotional interpretation of care and perceived failure of communication. This makes family communication and expectation management central components of violence prevention [10-12].

Communication failure appears to be one of the most modifiable contributors to aggression. Poor explanation, rushed interaction, inconsistent information, lack of empathy, and failure to acknowledge relatives’ concerns can rapidly erode trust. However, the responsibility for communication quality should not be placed entirely on individual doctors. Effective communication requires supportive systems, including manageable workload, designated spaces for family discussion, structured update times, clear documentation, and institutional policies that protect doctors while ensuring that relatives receive timely information [13,14].

System-level contributors are equally important. Overcrowding, lack of beds, shortage of medications, delays in imaging or laboratory services, weak security systems, and poor complaint mechanisms can all increase relatives’ frustration. In resource-limited hospitals, doctors may become the visible face of system failure even when they have little control over the underlying problem. This is particularly relevant to African and low-resource settings, where healthcare workers may face high clinical demand with limited institutional protection and weak reporting systems [3,4].

The geographical distribution of the evidence base should also be interpreted cautiously. Several primary burden studies cited in this review originate from Asian healthcare systems, including China, Bangladesh, and India, while African evidence is mainly represented by a continental systematic review and selected emergency department data from Egypt. Therefore, findings from South and East Asian tertiary hospitals should not be assumed to directly represent African or Sudanese hospital settings. Differences in hospital infrastructure, staffing levels, security arrangements, reporting culture, health literacy, patient flow, and relatives’ expectations may substantially influence both the frequency and form of aggression. For this reason, regional claims in this review should be understood as contextual rather than definitive, and the findings support the need for locally generated primary data from African and Sudanese hospitals.

This review differs from prior broad workplace violence reviews by focusing specifically on patients’ relatives and companions as a distinct contributor to aggression against doctors. Rather than repeating prevalence summaries alone, the review integrates primary hospital-based evidence with an organizational and patient-provider conflict framework to explain how system pressure, relatives’ emotional responses, communication failure, and weak institutional prevention capacity interact.

An important limitation of the available evidence is that many cited studies originate from a limited number of countries and healthcare systems. Therefore, findings should not be generalized without considering regional differences in hospital infrastructure, staffing patterns, patient flow, security systems, reporting culture, health literacy, and expectations of relatives. For example, the drivers of aggression in well-resourced emergency departments may differ from those in resource-limited tertiary hospitals, where shortages of beds, medications, investigations, and staff may intensify waiting times and dissatisfaction. These contextual differences limit external validity and support the need for locally generated data, particularly from African and resource-limited hospital settings.

The consequences of violence extend beyond the affected doctor. Repeated exposure to aggression can lead to burnout, anxiety, reduced morale, defensive medical practice, and avoidance of difficult communication. At the team level, violent incidents disrupt workflow, reduce trust, and affect the overall safety climate. At the organizational level, workplace violence contributes to underreporting, staff dissatisfaction, absenteeism, and poor retention. These effects demonstrate that violence against doctors is not only an occupational hazard but also a threat to patient safety and healthcare quality [16,17].

Prevention strategies, therefore, need to be integrated and practical. Communication training, de-escalation skills, family update systems, visible triage explanations, incident reporting tools, security protocols, and leadership accountability should function together rather than as isolated interventions. In resource-limited settings, prevention does not always require expensive infrastructure. Low-cost strategies such as clear signage, structured family counseling, waiting-time explanations, staff training, and simple reporting forms can help reduce uncertainty and identify repeated triggers for aggression [18,19].

Overall, the evidence supports a shift from reactive violence management toward proactive violence prevention. Hospitals should identify high-risk settings, strengthen communication systems, support staff after incidents, and collect reliable data on violent events. Future research should give greater attention to patients’ relatives as a distinct group rather than merging them with patients or visitors. More studies are also needed from African and resource-limited healthcare systems to understand how local cultural expectations, hospital infrastructure, and resource shortages shape aggression toward doctors [3,10].

Limitations of this review

This review has several limitations. First, as a narrative review, the selection and interpretation of sources may be influenced by narrative selection bias, and the findings should not be interpreted as a systematic or exhaustive synthesis of all available evidence. Second, only English-language sources were included, which may have introduced language restriction bias and excluded relevant studies published in other languages. Third, the search was conducted across selected databases and scholarly platforms, which may have introduced database selection bias. Fourth, no protocol registration, Preferred Reporting Items for Systematic Reviews and Meta-Analyses (PRISMA) flow diagram, formal risk-of-bias assessment, or pooled quantitative synthesis was performed, consistent with the narrative design of the review. Fifth, much of the evidence base comes from a limited number of countries and healthcare systems, with several primary studies originating from China, Bangladesh, India, and Egypt. African evidence is mainly supported by one continental systematic review and selected Egyptian emergency department data, while Sudanese primary data are lacking. Finally, several prevention recommendations are supported by expert consensus or rapid-review evidence rather than high-quality intervention trials, meaning that their effectiveness, feasibility, and cost should be evaluated locally before implementation.

This review highlights that reducing violence against doctors requires protecting healthcare workers while also improving the experience of patients and relatives. A balanced approach should acknowledge relatives’ distress without tolerating aggression, support doctors without blaming them for system failures, and strengthen hospital processes that promote trust, transparency, and safety. When communication, institutional preparedness, and staff protection are integrated, hospitals are more likely to reduce aggression and maintain safer clinical environments for both healthcare workers and patients.

## Conclusions

Violence against doctors by patients and their relatives is a significant and multifactorial problem in hospital settings. The available evidence shows that aggression toward doctors is driven by emotional distress, unmet expectations, communication failures, long waiting times, overcrowding, resource limitations, and weak institutional prevention systems. Patients’ relatives are particularly important because they often act as caregivers, decision-makers, financial supporters, and advocates during highly stressful clinical encounters. Communication failure remains one of the most important modifiable contributors, especially when diagnosis, prognosis, triage decisions, delays, and treatment plans are not clearly explained.

Reducing violence against doctors requires an integrated hospital-level approach that protects healthcare workers while improving communication, trust, and transparency with patients and relatives. Effective prevention should include staff training, structured family updates, visible triage and waiting-time explanations, environmental safety measures, incident reporting systems, leadership accountability, and post-incident support. Future research should focus more specifically on relatives and companions as a distinct group, particularly in emergency departments, tertiary hospitals, and resource-limited healthcare systems, to support context-appropriate strategies for safer hospital environments.
